# The bacterial community of childcare centers: potential implications for microbial dispersal and child exposure

**DOI:** 10.1186/s40793-022-00404-6

**Published:** 2022-03-04

**Authors:** D. E. Beasley, M. Monsur, J. Hu, R. R. Dunn, A. A. Madden

**Affiliations:** 1 Department of Applied Ecology, North Carolina State University, Raleigh, NC 27695 USA; 2 Department of Biology, Geology and Environmental Science, University of Tennessee Chattanooga, Chattanooga, TN 37403 USA; 3 College of Design, North Carolina State University, Raleigh, NC 27695 USA; 4 The Microbe Institute, Everett, MA 02149 USA; 5 Present Address: Department of Landscape Architecture, Texas Tech University, Lubbock, TX 79409 USA

**Keywords:** Built environment, Childcare centers, Early childhood education centers, Microbiome, Preschool children, Fomites

## Abstract

**Background:**

Bacterial communities within built environments reflect differences in sources of bacteria, building design, and environmental contexts. These communities impact the health of their occupants in many ways. Children interact with the built environment differently than do adults as a result of their unique behaviors, size, and developmental status. Consequently, understanding the broader bacterial community to which children are exposed will help inform public health efforts and contribute to our growing understanding of the bacterial community associated with childcare centers.

**Methods:**

We sampled childcare centers to survey the variation in bacterial community composition across five surfaces found inside and outside twelve classrooms and six centers using 16S rRNA marker gene amplicon sequencing. We then correlated these bacterial community analyses of surfaces with environmental and demographic measures of illumination and classroom occupant density.

**Results:**

The childcare environment was dominated by human-associated bacteria with modest input from outdoor sources. Though the bacterial communities of individual childcare centers differed, there was a greater difference in the bacterial community within a classroom than among centers. Surface habitats—fomites—within the classroom, did not differ in community composition despite differing proximity to likely sources of bacteria, and possible environmental filters, such as light. Bacterial communities did correlate with occupant density and differed significantly between high and low usage surfaces.

**Conclusions:**

Our results suggest built environments inhabited by young children are similar to functionally equivalent built environments inhabited by adults, despite the different way young children engage with their environment. Ultimately, these results will be useful when further interrogating microbial dispersal and human exposure to microorganisms in built environments that specifically cater to young children.

**Supplementary Information:**

The online version contains supplementary material available at 10.1186/s40793-022-00404-6.

## Background

People spend the majority of their lives in built environments, and thus the microbes dispersed to these habitats, and which in turn occupants are exposed to, are particularly relevant to human health. These microbe-occupant interactions can be benign, detrimental, or beneficial. Microbial communities on the surfaces of indoor environments—their diversity, their composition, and their activity—have been associated with public health outcomes directly in relation to microbial transmission (such as food poisoning and respiratory infections) as well as with more complex health outcomes such as asthma and allergies [1].

Bacterial communities of built environments are largely shaped by factors that influence the dispersal, recruitment, and persistence of bacteria. These include architectural design (e.g., ventilation [2]), building condition [3], outdoor environmental factors such as climate, vegetation, and light [4, 5], and diverse sources of bacteria (e.g., humans and dogs) [6, 7]. The behaviors of human occupants can also impact these communities, both across and within built environments. For example, kitchens and bathrooms of homes harbor distinct bacterial communities in part because of how occupants contribute to the dispersal of differing bacterial taxa in these spaces (i.e. through transfer from foods, versus waste processes) [6]. As another example, high and low contact surfaces in university classroom spaces have differing bacterial communities, likely as a result of differential dispersal of bacteria from human skin [8]. Even a child crawling on a surface can impact the bacteria aerosolized, which in turn impacts what bacteria they are exposed to [9, 10]. How occupants interact with the built environment dictates both microbial dispersal and exposure.

Preschool-aged children (ages 3–5 years) interact with one another and with the physical environment in a manner that differs from the interactions of adults due to the children’s smaller size, earlier developmental stages, more nascent motor skills and unique behaviors. For example, children tend to have a higher frequency of hand-to-mouth contact as well as mouth contact with various objects than do adults [11]. Furthermore, the first decade of life features somewhat predictable changes in the gut microbiome, and thus children may present unique bacterial sources of dispersal compared to adults [12]. Additionally, in many countries children now occupy different indoor spaces than those occupied by most adults, specifically childcare centers (alternatively referred to as early childhood education centers, preschools, daycares, nursery schools, and early learning centers). In such childcare centers, children can be exposed to a diversity (and abundance) of microbes with direct health implications, such as viruses, enteropathogens, and bacteria containing antibiotic resistance genes [13–17]. Understanding the broader bacterial community present in such environments therefore provides additional context for future school design and improved public health efforts [17–22].

Previous studies on the indoor microbial community of childcare centers have revealed a community greatly influenced by human occupancy and outdoor environmental sources, similar to other built environments [17, 22]. The community is typically dominated by human skin-associated bacteria such as taxa in the classes Gammaproteobacteria, Alphaproteobacteria, and Betaproteobacteria, as well as lactic acid bacteria and those in the genus Propionibacterium [19, 20]. While these community patterns recapitulate those observed in adult-associated spaces, the limited number of studies directly comparing built environments occupied by different age groups have observed age-associated differences. For example, a study comparing childcare centers (occupied by 5 year-olds) with elementary schools (occupied by 8–9 year-olds) in Seoul, South Korea, observed notable differences in these aerosolized bacterial community across spaces occupied by these different age groups [22]. Bacterial communities sampled within childcare centers had higher relative abundances of the skin-associated *Streptococcus* genus and feces-associated *Paracoccus* than found in elementary schools. These differences were observed in the inside of these classrooms, while no difference was found in the outdoor bacterial communities of these schools.

While the bacteria of childcare centers reflect the microbiomes, behaviors, and contexts of the occupants, the fungi in these environments often reflect environmental sources and determinants. Sources of fungi in the indoor environment include building materials, ventilation systems, human transmission (i.e. through fungi tracked into the building from environmental sources), and the greater outdoor environment [21, 23], with indoor fungal communities in childcare centers dominated by the genera *Aspergillus, Cladosporium, Penicillium, Alternaria, Rhizopus and Curvularia.* High abundances of these fungi in built environments have been associated with various respiratory disorders such as asthma and allergic rhinitis [24, 25]. Because of the health consequences of microbial exposure—be it bacteria, fungi or other taxa–to young children, it is important to better characterize the indoor microbial community in the context of their unique behaviors and interactions with the built space [26, 27].

This study considers the bacterial community in preschool child-associated spaces with a particular focus on understanding differences in communities across high and low contact surfaces, in comparison with past studies which have concentrated on the bacterial communities of floor dust (e.g. [17]), or air (e.g., [19, 20, 22, 28]); but see [18]. First, we ask how bacterially unique classrooms are, one to the next, by comparing the bacterial communities across surfaces in classrooms and childcare centers in the Raleigh-Durham area of North Carolina in the United States of America. This allowed to us to compare multiple centers while reducing the variation expected from comparing indoor environments across broader geographies [29]. We then consider the extent to which the bacterial communities differ within classrooms, specifically with regard to human-associated taxa, by comparing locations within the interior of the classroom and immediately on the exterior of the classroom building. Additionally, we test whether the bacterial community composition within classrooms reflect the level of use within the classroom by comparing high-contact surfaces (surfaces that are more often touched and more often cleaned) and low-contact surfaces. Finally, we examine environmental illumination and child occupancy differences across sampling locations and classrooms that may contribute to differences in bacterial communities.

## Methods

### Sampling design

**Fig. 1:**
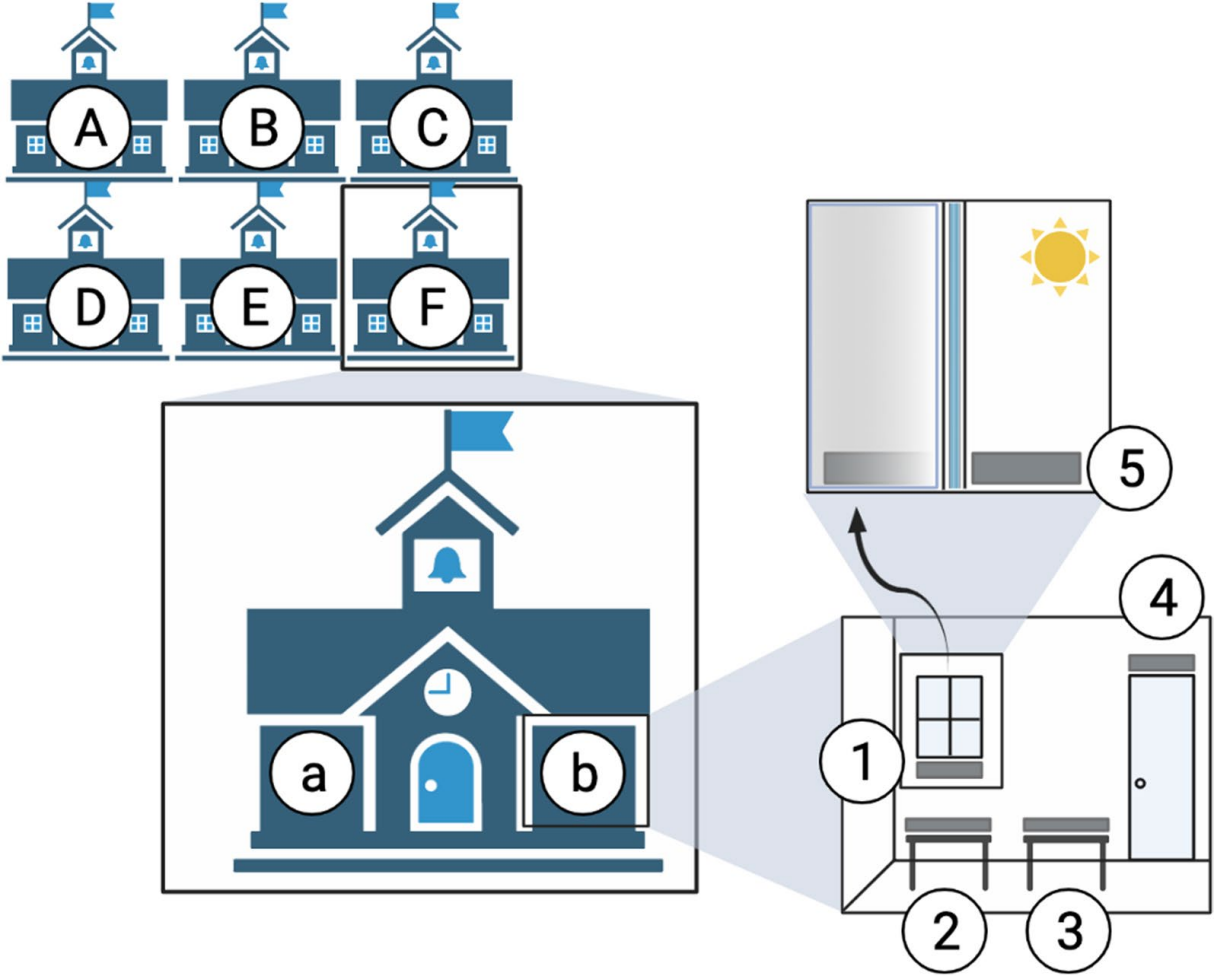
Bacterial sampling schema of childcare centers. Two classrooms (“a” and “b”) were sampled from each of six childcare centers (“A-F”) in the Raleigh-Durham area of North Carolina. Samples from each classroom included: “1.” Inside window, “2.” Desk surface near window, “3.” Desk surface near door, “4.” Inside classroom door trim of door to interior of building, and “5.” Outside window of classroom. Image created with BioRender.com

We selected six childcare centers within the Raleigh-Durham area of North Carolina for bacterial community sampling. The childcare centers served preschool children 2.5–4 years in age. Classroom sizes varied from 300 to 1400 square feet and student occupancy ranged between 7 and 20 students. Within each school we sampled two classrooms (Fig. 1).

We sampled five locations (habitats) within each classroom using a dual-tipped sterile BBL™ CultureSwabs™: interior window sill, desk surface near a window, desk surface near the interior classroom door, upper door trim on the interior of classroom, and exterior window (Fig. 1). We selected these locations because they are common across classrooms and they vary in degree of human contact—both in terms of how often they are touched by children and in terms of how often they are cleaned. In as much as inside door trim is both rarely directly touched and cleaned we used it here, as elsewhere [4, 6] as a measure of the general cloud of airborne bacteria that settle on surfaces. We sampled the exterior and interior window sills to compare outdoor bacterial source communities to classroom bacterial communities [5, 8]. As visible light has been found to shape dust bacterial communities [5, 6, 30], illuminance levels were recorded for each sample location using LI-COR sensors. Sampling was conducted in duplicate, once in fall 2014 and once in spring 2015, although only spring samples were used in downstream analyses (see below).

### Amplicon preparation and sequencing

Dry swabs were collected in ambient conditions and then brought back to the lab within 0.5 h and stored in a − 20 °C freezer. Samples were then shipped to the University of North Carolina Microbiome Core Facility on ice, where DNA amplification, library preparation, and sequencing were carried out. Briefly, DNA was extracted from the swabs using a commercial DNA isolation kit (Qiagen, Valencia, CA) per the manufacturer’s protocol. The 16S ribosomal gene region V1-V2 was amplified (forward primer: AGAGTTTGATCCTGGCTCAG, reverse primer: GCTGCCTCCCGTAGGAGT) with PCR cocktails containing 50 ng of DNA template, HotStar Hi-fidelity DNA polymerase (Qiagen, Valencia, CA), HotStar Hi-Fidelity PCR buffer with dNTPs, and 0.4 µM of each primer. The cycling parameters were 35 cycles of 60 s 94 °C, 60 s 50 °C, and 60 s 72 °C, followed by a final extension at 72 °C for 10 min. Libraries were prepared by purifying the amplicons using the AMPure XP kit (Beckman Coulter) and then barcoding the amplicons with 10–12 basepair sequence tags for sequence multiplexing. Samples, a sterile swab, and no template controls were run on two runs on the Ion Torrent PGM platform, using 318 chips with 200/400 single-read sequencing [31].

### Sequence processing

Sequencing resulted in 7,161,237 reads for the spring samples and 6,763,698 reads for the fall samples. Sequences were processed using the UPARSE [31] and QIIME [32] pipelines. Synthetic adapters were removed using cutadapt (1_8_1) [33]. Sequences were quality filtered in USEARCH7 (v7_0_1) with a maxee of 1.5 (allowing for 1.5 sequence errors per read). Sequences were then demultiplexed in QIIME (1_9_1), and further filtered to have a minimum length of 100 base pairs. This resulted in 1,375,539 sequences. Sequences from the two Ion Torrent sequencing runs (fall and spring sampling) were subsequently processed together, and sequence headers were modified from QIIME format to UPARSE formats using a modified version of the Brazilian Microbiology Protocol’s custom script [34]. Sequences were dereplicated and singletons were removed. De novo clustering was performed at 97% using USEARCH7, along with chimera checking. The initial representative set of sequences from these clusters was filtered against the Greengenes database (13_8) [35] at > 75% to remove non-target sequences. This filtering resulted in the removal of 8.9% of the OTUs. The quality filtered and dereplicated sequences were mapped back to this reference database at 97% (1,138,469 sequences successfully mapped). Taxonomy was assigned using the Ribosomal Database Project naïve Bayesian classifier [36] with a confidence threshold of 0.5, against the Greengenes database (13_8) [35]. Reads originating from chloroplasts and mitochondria were removed (leaving 767,248 sequences). As these were extremely low biomass samples, we took the conservative approach of removing all OTUs that were present in the negative control samples. These negative control samples included an unused “blank” swab (for extraction and sampling control) and a reagent only control. Twenty-one OTUs were detected in these negative controls and represented only 2.9% of the total sequences (a full list of these OTUs and their taxonomy is presented in Additional file 1: Table S1) OTUs in control samples were removed from all samples (leaving 744,698 sequences). Manual BLAST comparisons [37] of OTUs with poorly resolved taxa (resolved only to kingdom) against the NCBI database revealed low similarity values, suggesting non-target sequences. These OTUs were removed from the dataset, resulting in 743,073 sequences in 118 samples (min = 4, median = 5490, max = 57,785 sequences per sample) and a total of 5,205 OTUs. Due to concerns about run-to-run variation, only samples from the spring season of sampling (April) were included in this study. This resulted in 218,737 sequences and 59 samples (min = 4, median = 3906, max = 8,741 sequences per sample, total OTUs = 5205). Samples were rarefied at 200 sequences per sample, to incorporate as many of the samples as possible in the community analyses. This resulted in 2,801 OTUS across 57 samples (outside, exterior window: N = 10, inside window: N = 12, inside desk by a window N = 12, inside desk by the classroom door N = 11, and inside classroom door trim N = 12). Two hundred sequences per sample is a relatively low sequencing depth for such community composition comparisons, therefore we confirmed the robustness of our analyses by additionally performing all correlation analyses and community diversity analyses with another dataset where samples were rarefied at 1000 sequences per sample. Sequencing depth had no substantive impact on our results (Additional file 1: Table S2), therefore, we present the results using the 200 sequence depth—with a corollary higher sample number—herein.

To determine if locations within the classroom varied in the proportion of dispersed bacteria from windows (as a potential source of environmental bacteria), we used a second dataset that included the relative contribution of chloroplast DNA to a given sample (as a proxy for pollen deposition). We generated this dataset by including the total sequences minus those assigned to mitochondria, plus those sequences assigned to chloroplasts [17]. This dataset was also rarefied to 200 sequences per sample prior to analysis.

### Statistical analysis

We conducted all statistical tests in R [38]. Bacterial communities were compared by calculating beta diversity using a Bray–curtis dissimilarity metric on square-root transformed data using the MCTOOLSR package [39].

To determine if the bacterial community outside of childcare classrooms differed significantly from the communities inside, we broadly compared inside window sill samples with outside window sill samples (inside window: N = 12, outside window: N = 10). We calculated differences in community diversity (beta diversity) using PERMANOVA (adonis) tests in the VEGAN package with 999 permutations [40]. To determine if these differences in inside/outside communities differed by childcare center, we compared samples with classroom and center as fixed effects in our model and performed a second analysis with samples blocked by the factor center. All analyses were in alignment, so we report the results of the blocked analysis in the text. To determine if outside versus inside samples differed in community membership (alpha diversity) we calculated bacterial observed species in the MCTOOLSR package [39] and compared them across inside window and outside window environments across classrooms with a Kruskal–Wallis test in the STATS package [38].

To determine if communities inside classrooms varied by classroom and center we pooled the samples of door trim (N = 12), desks (N = 23), and inside windows (N = 12) with classroom and centers as fixed effects in the model. We included the same inside samples when comparing bacterial communities across surfaces sampled, comparing by sampling location (unique habitats within a classroom). For comparisons of bacterial communities by surface usage we compared “high contact” desk samples (N = 23) with “low contact” door trim samples (N = 12) across classrooms. All beta diversity comparisons were made with adonis tests in the VEGAN package [40] using 999 permutations.

To determine what bacterial taxa might be driving differences in community composition across inside/outside samples and across high and low usage surfaces, we performed Kruskal–Wallis tests comparing average relative abundances of bacterial genera and families, then corrected for multiple comparisons using an FDR p-value correction using the package MCTOOLSR [39] per the methods used in Leff and Fierer (2013) and Dunn et al. (2013) [6, 41]. These taxon-based comparisons average the OTU abundances by the specified taxonomic level, thus reducing the number of comparisons (and the spurious statistical results that can result from such multiple comparisons). We supplemented these analyses with a DESeq2 negative binomial Wald test in MacQIIME (running QIIME 1_9_1 [32] and the R package DESEQ2 [42]) followed by Benjamini–Hochberg FDR post hoc corrections to determine what OTUs may be differentially abundant across sample types. For this analysis, we used a sequence dataset that was non-rarefied and removed all samples with less than 1000 sequences per sample. This dataset included all 5,205 OTUs following this filtering step. We further removed rare OTUs (those with less than 0.1% per sample). These filtering steps are required because the DESeq2 dispersion analysis is sensitive to small mean counts. This filtering resulted in 30 samples for the high versus low contact surface comparison (high contact, N = 19, low contact, N = 11), with 185 OTUs (sequences counts per sample: min = 1432, median = 2463, max = 7149). Diagnostic analyses of the dispersion of these data revealed this was not an appropriate test for the dataset comparing outside and inside samples (data not shown), so this method was only used to compare high contact versus low contact surface communities.

To determine if environmental and occupancy factors contributed to differences in classroom bacterial communities, we calculated dissimilarity matrices of lux (square root transformed per location) and occupant density (children/square foot classroom per classroom) using the Bray–curtis method in the ECODIST package [43]. We then determined if these factors correlated with changes in bacterial communities (beta diversity) by calculating independent mantel correlation statistics based on Pearson’s product-moment correlation in the VEGAN package [40] using 999 permutations.

To understand if distance from possible dispersal sources (e.g., windows) was contributing to bacterial community composition, we used a second rarefied dataset that included sequences associated with chloroplasts as a proxy for pollen deposition. We compared the relative abundance of chloroplast sequences across sample locations (across classrooms) using a Kruskal–Wallis test with posthoc Dunn tests using the STATS and PMCMR packages [44].

We determined if occupant density significantly varied by center, thus presenting a lurking variable in our correlation analyses, by comparing occupant density by center using a Kruskal–Wallis tests with post-hoc Dunn tests using the STATS and PMCMR packages [44]. We used the same calculations to determine illumination (Lux) exposure differences across sample locations.

We visualized relative abundance of taxa across sampling locations by creating heat maps in MCTOOLSR [39] scaled by sampling location or taxon. We calculated and visualized ordination plots (nonmetric multidimensional scaling plots) in MCTOOLSR [39] and illumination, occupancy, relative abundance of taxa, alpha diversity, relative abundance of chloroplast sequence reads, and significantly enriched OTUs in high contact surfaces using the GGPLOT2 package [45].

## Results

### Human-associated taxa drive differences in the bacterial community of childcare centers between inside and outside samples

**Fig. 2:**
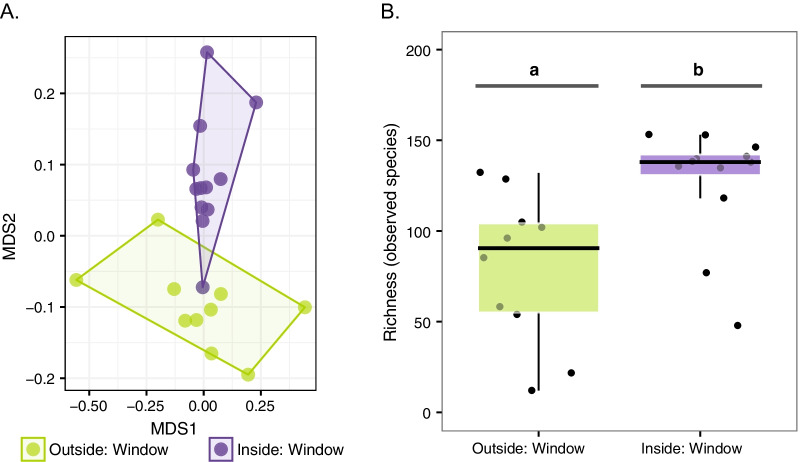
The bacterial communities of inside versus outside windows in childcare classrooms in North Carolina across classrooms. Bacterial community varied across inside and outside window locations as visualized with an ordination plot (NMDS) (**A**), and bacterial diversity (membership) varies as measured by species richness (**B**). Lowercase letters indicate statistically significant differences

Consistent with other built environment studies, including those involving childcare centers, the bacterial communities of childcare centers differed significantly from the bacterial communities outdoors, even though outdoor samples were from outside window sills separated by just inches from the classroom window samples inside (Fig. 2A) (across classrooms, adonis, R^2^ = 0.10, *p* = 0.001).

In addition to differences in community composition, the inside and outside samples differed in species diversity. The bacterial communities on the inside windowsills were significantly more diverse than those on the outer window sills (median observed species: 138, range: 48–153, versus median observed species: 90.5, range 123–132) (Kruskal–Wallis, *p* < 0.01) (Fig. 2B). This follows the same trend seen in house dust when inside communities are compared with those of outside communities [4].

**Fig. 3:**
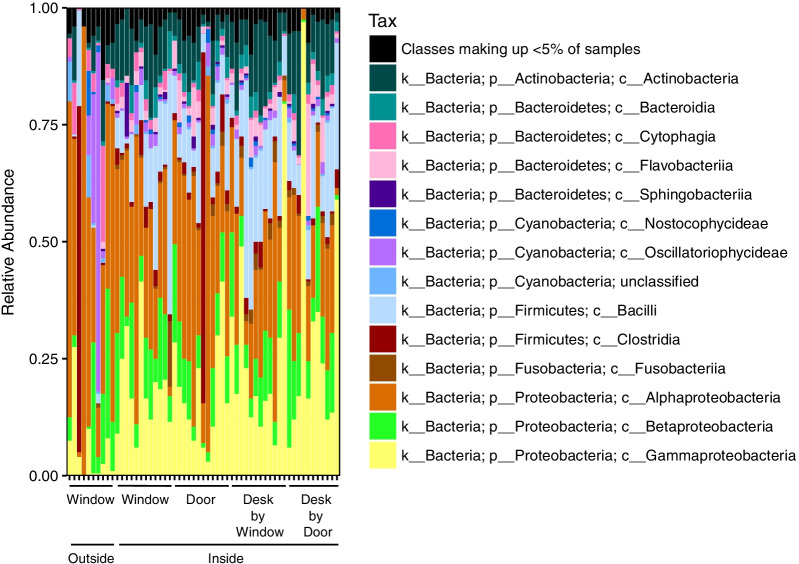
The relative abundance of sequences from various bacterial classes across locations inside and outside of classrooms (across childcare centers)

**Fig. 4:**
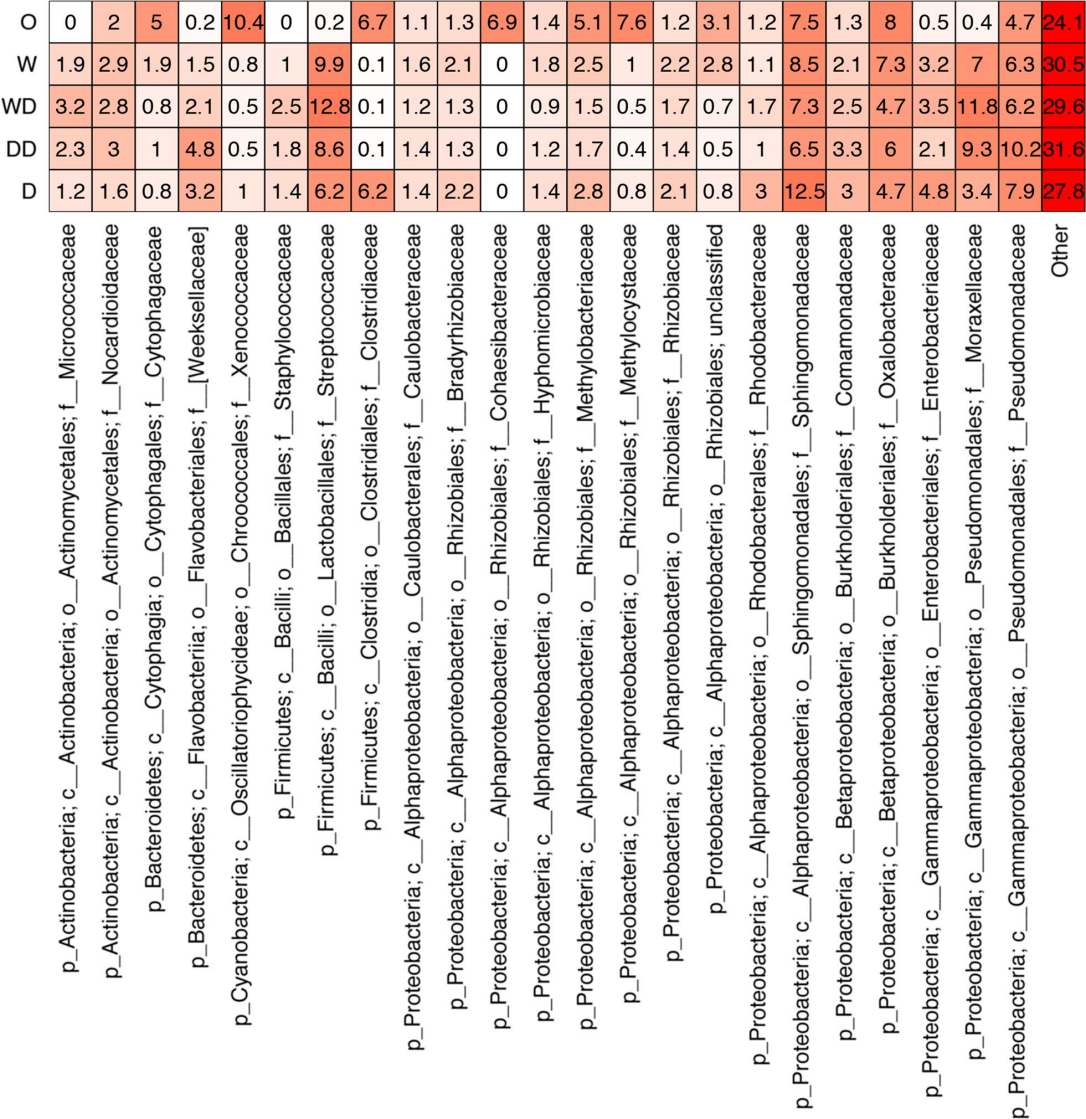
Heatmap of the relative abundance of the 23 most abundant families detected across sampled locations. “O” = outside window, “W” = inside window, “D” = inside door, “DD” = inside desk near door, “WD” = inside desk near window. Color is scaled by location

The outside communities included taxa consistent with environmental sources, such as cyanobacteria (*Xenococcaceae), Methylobacterium* spp., species of the order *Rhizobiales*, and *Pseudomonas* spp., whereas inside window communities contained these species in addition to those taxa typically associated with human bodies (Figs. 3, 4).

Specifically, inside window samples were dominated by significantly higher relative abundances of sequences from bacterial families associated with skin and oral cavities. These included Moraxellaceae (e.g. *Acinetobacter* spp.) (Kruskal–Wallis, adjusted *p* < 0.001), and Streptococcaceae (e.g. *Streptococcus* spp.) (Kruskal–Wallis, adjusted *p* = 0.001) (Fig. 4).

### Locations within a classroom are more variable than across centers, with surface activity driving differences in bacterial communities

**Fig. 5:**
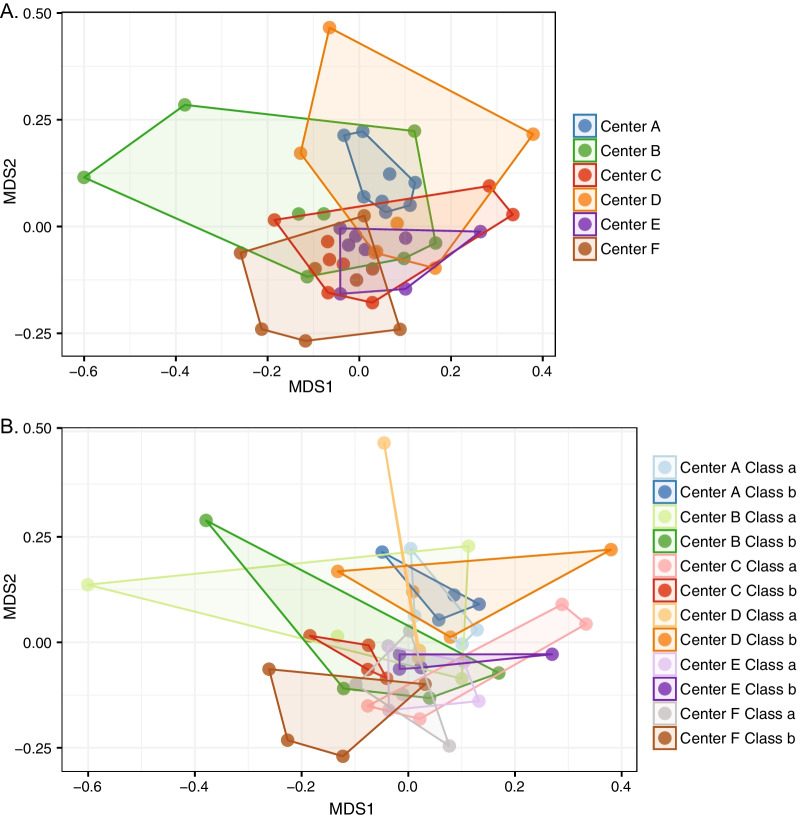
Bacterial communities varied across classrooms and childcare centers. NMDS ordination plots of the bacterial communities of all inside samples as a function of centers (**A**), and classrooms within centers (**B**). Centers = “A-F”, Classrooms per center = “a” and “b.”

Bacterial communities inside childcare centers significantly differed across centers (adonis, R^2^ = 0.149, *p* = 0.001), and classrooms (adonis, R^2^ = 0.141, *p* = 0.005) (Fig. 5).

These differences in bacterial communities were significantly correlated with the density of occupants as measured by the number of children in a class per square foot of classroom space (Mantel, Rho = 0.19, *p* = 0.04) (Additional file 1 Table S3). However, caution should be used when attributing causation to this correlation, given that occupant density varied by centers (though not significantly (Kruskal–Wallis, *p* > 0.05) (Additional file 1 Fig. S1)). While some individual classrooms had unique bacterial communities, samples were often less similar among locations within a given classroom, than among centers (Figs. 3, 5). Therefore, this correlation could be driven by factors that differed between centers that we did not capture in our study.

Sampling locations within a classroom experienced a predictable gradient of illumination, with locations nearer the window experiencing significantly greater illumination than those near the inside classroom door (Kruskal–Wallis, *p* < 0.001) (Additional file 1: Fig. S2). Indeed, inside window samples received only 6% of the illumination of outside samples, and door trim samples received less than 1%. These differences in illumination among locations suggest the locations sampled are across a similar distance gradient from a possible bacterial source population (i.e. the window). Despite this, the relative abundance of 16S rRNA chloroplast sequences (a proxy for pollen deposition from windows) did not significantly vary across inside locations (Kruskal–Wallis, *p* > 0.05) (Additional file 1: Fig. S3). Bacterial communities within a classroom, similarly, did not significantly vary by location despite experiencing differences in illumination (adonis, *p* > 0.05). Even locations closer to the window (and presumably more exposed to outdoor bacterial inputs) were no more like the outdoors than were habitats far from the windows (Additional file 1: Fig. S4).

All samples within a classroom reflected the patterns observed in the inside window samples, and patterns observed in classroom and home built environments [5, 7, 8], including the presence of environmental species in the genera *Pseudomonas*, *Methylobacterium*, and *Janthinobacterium*, and an abundance of human-associated bacterial taxa. Often these human-associated bacteria dominated samples. For example, the single skin and oral cavity-associated genus *Streptococcus* accounted for more than 5% of the sequences in indoor habitats across surfaces sampled (Additional file 1: Fig. S5), and made up nearly 1/5^th^ of the bacterial communities of four individual desks across four centers. Similarly, 9.5% of the bacterial community of one sample was composed of the gut-associated *Trabulsiella* genus, while two individual desk samples contained communities where nearly half the sequences came from the often oral cavity and skin-associated *Acinetobacter* genus.

**Fig. 6:**
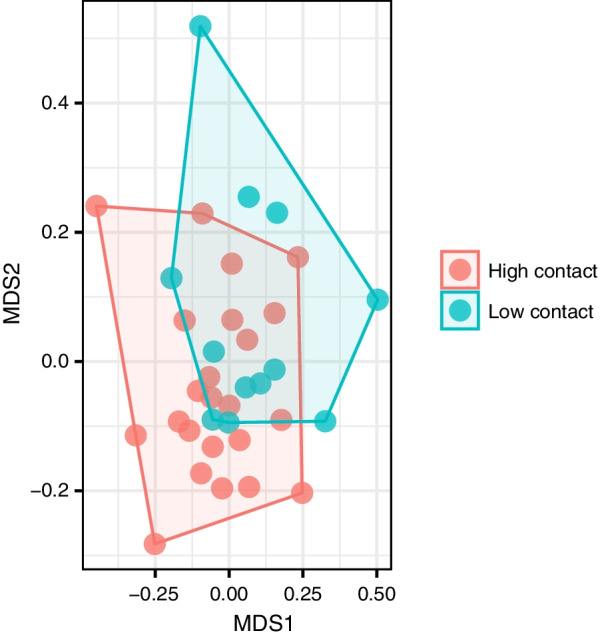
Bacterial communities varied by surface usage. NMDS ordination plot of the bacterial communities of inside classroom samples as a function of usage. Desk samples were grouped as “High contact” surfaces, while inside door trim samples were compared as “Low contact” surfaces

Bacterial communities within classrooms did not differ by environmental factors such as illumination, which has been found to shape the living and dead bacterial communities of indoor samples [5]; however, these communities did significantly vary by surface usage (adonis, R^2^ = 0.04, *p* = 0.005) (Fig. 6).

These community differences were partially driven by an increase in the air-associated genus *Sphingomonas,* which was significantly more abundant in low contact, door trim surfaces (Kruskal–Wallis, adjusted *p* = 0.01), and higher abundances of the often skin-associated Moraxellaceae (e.g. *Acinetobacter* spp.) in high contact, desk surfaces (Kruskal–Wallis, adjusted *p* = 0.04) (Additional file 1: Fig. S5). These results were supported by the DESeq2 analysis of differentially enriched OTUs among habitats, which revealed OTUs assigned to skin and saliva associated taxa (e.g., Streptococcaceae*,* Micrococcaceae, and Moraxellacceae) were significantly more abundant in high contact surface communities (adjusted *p* < 0.05) (Additional file 1: Fig. S6).

## Discussion

Microbial exposure in built environments impacts health, mediated by complex relationships with the hosts behaviors (e.g., time of exposure, frequency of contact, quality of interaction), host immune system (e.g., occupant immune system development stage, health status), and the physiology of the microbes in question (e.g., viable or non-viable, endotoxin-producing, and/or pathogenic, benign, or beneficial). The potential importance of microbial exposure to children, and the unique relationships children have with their environment, lends further importance to understanding the broader microbial communities of child-dominated spaces. Here, in six childcare centers, comprising twelve classrooms in North Carolina in the United States of America we found habitats within classrooms dominated by a relatively small subset of human-associated bacteria highlighting the importance of high touch surfaces in dispersal and exposure.

Consistent with previous studies, the inside environment differed from the local outside bacterial communities, both with regard to the identity of bacterial taxa and their likely sources. Inside bacterial communities consisted primarily of skin and oral-associated bacteria with additional outside inputs. Outside samples reflected the bacterial communities often observed on similarly exposed surfaces (e.g., tombstones, exterior house door trim) [4, 46], suggesting a greater input from environmental sources. These differences between inside and outside—both in species composition and diversity—are not surprising and are similar to that noted for houses [4], elementary schools [47], and childcare centers [17, 22].

We found a significant difference in bacterial communities between high and low contact surfaces, but not among bacterial communities experiencing different levels of visible light, nor distances from windows (as likely sources of environmental bacteria). Our data suggest the bacterial communities present in a classroom are therefore influenced by actions in the classroom in such a way as to lead different surface types having different bacterial communities. The bacterial communities in inside dust are known to be directly influenced by the number and types of occupants in the building [4]. In keeping with these findings, we found occupancy, or the density of children per classroom, correlated with differences in the bacterial communities of these spaces. The density of students, and thus the higher number of humans as microbial sources may drive the differences we noted in communities across classrooms. A recent study investigating the bacterial community of floors in 499 elementary school classrooms similarly found the number of students per classroom to correlate with differences in bacterial communities [47], a pattern also observed in other bacterial studies of homes [48]. Our findings are aligned with those of other studies of buildings occupied primarily by adults, which found bacterial composition varied among surfaces (fomites), due to how often they were touched or likely to be cleaned [6, 8], or other factors [2, 49].

Studies on childcare centers show that the indoor microbial community is similar to adult spaces in that the community is dominated by microbes associated with human skin [17, 20, 22, 50, 51]. Like most previous studies of childcare centers, we found that fecal bacteria were not as abundant in most inside surfaces as were those taxa associated with oral and skin habitats. Similarly, we found that the indoor microbial community was shaped primarily by human occupancy and activity and less by distance from environmental sources. In our study, these effects of occupancy were observed by comparing spaces that were used less or more often or by fewer or more people. The same patterns seem apparent in studies through time. For example, Nygaard and Charnock sampled floor dust samples from a newly opened kindergarten and showed a change in the indoor bacterial community over time, which reflected the changes in human occupancy (adult vs. pre-pubescent children) [19].

The techniques used in this study measured the relative abundance of bacterial taxa, regardless of the viability, or ‘culturability’ of these strains. They therefore overcome certain limits imposed by culture-dependent studies and generally allow us to compare findings with other broad-scale studies of comparable built environments; however, our chosen methods are blind to whether those bacteria detected are alive or dead. These techniques similarly don’t allow us to understand absolute abundance, or pathogenicity of any given bacterial taxon. It may be that bacterial communities on high contact surfaces are dead, as many of the microbes on surfaces in built environments are non-viable [52]. However, there are implications of these findings beyond the direct effects of beneficial or deleterious viable bacteria. Nearly half of the sequences in any sample come from gram negative bacteria (and in at least one sample made up the entire community). Regardless of viability, gram negative bacteria contribute pro-inflammatory endotoxins (lipopolysaccharides) to the environment. Inhaled endotoxins can contribute to inflammation associated disease states [23, 53]. Conversely, there is intriguing data to suggest exposure to specific bacterial taxa (e.g., *Mycobacterium vaccae*, various species of *Lactobacillus* spp. and *Bifidobacteria* spp., *Clostridium butyricum*, and *Bacteroides fragilis*), and even endotoxins may have positive therapeutic effects in certain circumstances [54, 55], even when these bacteria are not alive [56]. While these exposure outcomes can present an opportunity for concern or enthusiasm, future studies are required to assess both absolute exposure of these bacteria (and their byproducts) to children in childcare centers and the consequences of this exposure given the unique ways children interact with their built environment [57].

Prior research focused on fungi in the built environment has revealed the potential health impacts of fungi, regardless of microbial viability, on human occupants. Indoor fungal community has been associated with the onset and/or worsening of respiratory illness, asthma, rhinitis, eczema, and allergic alveolitis [23, 58–60]. Fungi may cause negative health outcomes through interaction with their components (i.e. glucans), through the production of metabolites [61, 62], or through facilitating the growth and transmission of other microbial pathogens in the built environment [63]. Thus, understanding the indoor fungal diversity could further our understanding of age-specific exposure pathways and transmission. For example, a recent study showed that less-occupied rooms in childcare centers were significantly different from the main rooms in fungal diversity, [21]. These findings, in addition to our own, have implications for understanding age-specific exposure to microbes and fungi and their different health outcomes [64], and highlights why studies on the microbiome of the built environment occupied by different age groups are important.

A limitation of our study is our focus on a relatively small number of samples from classrooms in one city in the southeastern United States. While our findings are consistent with similar studies that investigated university classrooms, elementary schools, and childcare centers in other countries [8, 17, 22], there are limits to what we can assert based on the sample sizes in this study. Therefore, future studies are needed to understand the relationship of preschool age children and their built environments. Such studies would benefit particularly from pairing behavioral data on the frequency of contact with surfaces with these microbial studies, and including best sequencing practices (such as sequencing a mock community to assure results can be compared across studies as accurately as possible). This may ultimately inform our understanding of how different age groups engage with their specific environments while providing new insights into the relationship between the microbes of built environments and occupants.

## Conclusion

Past research has revealed that built environments show similar patterns in bacterial communities but are distinct based in part on occupants and the behaviors of these occupants in their environment. Consistent with previous studies on childcare centers, our research reveals that the childcare environment is similar to built environments such as homes and university classrooms, sharing dominance with human-associated taxa and with high-touch surfaces containing a greater proportion of human-associated bacterial taxa than low-touch surfaces. What emerges is that this childcare center bacterial community may not be driven by significantly different factors from functionally equivalent spaces (e.g. classrooms); however, the occupants of this environment will engage with the environment differently in ways that may impact exposure more than dispersal. As we are learning to engineer our environments to better control the microbial exposure of adult occupants (e.g. [2]), it is critical to investigate the unique relationships that exist between these communities and the occupants, particularly for built environments used in unique ways, and for those that may have unique occupant communities.

## Supplementary Information

**Additional file 1. Figure S1**. Occupancy of classrooms sampled in this study. Occupant density was calculated asthe number of children in the class per square foot of classroom space. Each point represents a unique classroom (n=2 per center). Occupant density did not significantly differ across childcare centers.** Figure S2**. Measures of illumination (lux) at sampling locations across classrooms. “O” =Outside Window, “W” Inside Window, “WD” Inside Desk near Window, “DD” Inside Desk near Door, “D” Inside Door. Lowercase letters indicate statistically significant differences in illumination among samples.** Figure S3**. Relative abundance of sequence reads assigned to chloroplast 16S in each sample based on location inside, or outside (building exterior) of classrooms. “O” = outside window, “W” = inside window, “D” = inside door, “DD” = inside desk near door, “WD” = inside desk near window. Lowercase letters indicate statistically significant differences among samples.** Figure S4**. Bacterial communities of surfaces varied by inside/outside location, but not by inside location. NMDS ordination plot of the bacterial communities of inside samples as a function of location. Inside: Desk (D) is the desk nearest the inside classroom door, while Inside: Desk (W) is the desk nearest the window.** Figure S5**. Heatmap of relative abundances of the 20 most abundant genera detected across sampled locations. “O” = outside window, “W” = inside window, “D” = inside door, “DD” = inside desk near door, “WD” = inside desk near window. Color scaled by genus.** Figure S6**. Differentially enriched OTUs in high contact surface communities (desks, n=19) versus low contact surface communities (door trim, n=11) across classrooms as determined by a DESeq2 negative binomial Wald test with FDR corrections. Twenty-three OTUs were significantly enriched across sample types. Notably, OTUs assigned to taxa frequently found in human saliva and skin (e.g., taxa in the families Streptococcaceae, Micrococcaceae, and Moraxellaceae) were enriched in bacterial communities on high contact surfaces.** Table S1**. List of putative contaminant OTUs found in the negative control samples that were removed from the original dataset.** Table S2**. Table of statistical results at different sequencing depths per sample.** Table S3**. Mantel tests based on Pearson’s product-moment correlation of Bray-Curtis distance metrics of lux and child occupant density for inside samples (inside window, desk by window, desk by door, inside door trim) across classrooms (samples: n = 47).

## Data Availability

The sequences and mapping files generated during the current study are provided in Figshare repository, https://figshare.com/s/57f7cca6e4d2ebedeeb2.
