# Supplementary material for: The bacterial community of childcare centers: potential implications for microbial dispersal and child exposure
Source: Environ Microbiome. 2022 Mar 4;17:8. doi: 10.1186/s40793-022-00404-6 (PMC8895594; doi:10.1186/s40793-022-00404-6)
Supplement: Supplementary file 1 — Additional file 1. Figure S1. Occupancy of classrooms sampled in this study. Occupant density was calculated asthe number of children in the class per square foot of classroom space. Each point represents a unique classroom (n=2 per center). Occupant density did not significantly differ across childcare centers. Figure S2. Measures of illumination (lux) at sampling locations across classrooms. “O” =Outside Window, “W” Inside Window, “WD” Inside Desk near Window, “DD” Inside Desk near Door, “D” Inside Door. Lowercase letters indicate statistically significant differences in illumination among samples. Figure S3. Relative abundance of sequence reads assigned to chloroplast 16S in each sample based on location inside, or outside (building exterior) of classrooms. “O” = outside window, “W” = inside window, “D” = inside door, “DD” = inside desk near door, “WD” = inside desk near window. Lowercase letters indicate statistically significant differences among samples. Figure S4. Bacterial communities of surfaces varied by inside/outside location, but not by inside location. NMDS ordination plot of the bacterial communities of inside samples as a function of location. Inside: Desk (D) is the desk nearest the inside classroom door, while Inside: Desk (W) is the desk nearest the window. Figure S5. Heatmap of relative abundances of the 20 most abundant genera detected across sampled locations. “O” = outside window, “W” = inside window, “D” = inside door, “DD” = inside desk near door, “WD” = inside desk near window. Color scaled by genus. Figure S6. Differentially enriched OTUs in high contact surface communities (desks, n=19) versus low contact surface communities (door trim, n=11) across classrooms as determined by a DESeq2 negative binomial Wald test with FDR corrections. Twenty-three OTUs were significantly enriched across sample types. Notably, OTUs assigned to taxa frequently found in human saliva and skin (e.g., taxa in the families Streptococcaceae, Micrococcaceae, and M [file 40793_2022_404_MOESM1_ESM.docx]

**Additional file 1: Supplemental Figures and Tables for the manuscript: “**The bacterial community of childcare centers: Potential implications for microbial dispersal and child exposure” by Beasley, D.E.^1,2^, Monsur, M., Hu, J., Dunn, R.R., and Madden, A.A.

**Figure S1.** Occupancy of classrooms sampled in this study. Occupant density was calculated as the number of children in the class per square foot of classroom space. Each point represents a unique classroom (n=2 per center). Occupant density did not significantly differ across childcare centers.


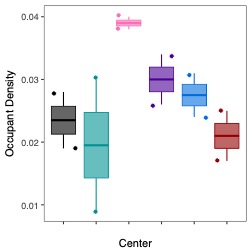


**Figure S2.** Measures of illumination (lux) at sampling locations across classrooms. “O” = Outside Window, “W” Inside Window, “WD” Inside Desk near Window, “DD” Inside Desk near Door, “D” Inside Door. Lowercase letters indicate statistically significant differences in illumination among samples.

**
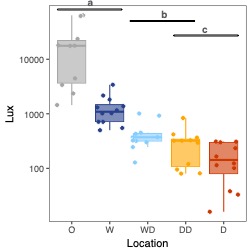
**

**Figure S3.** Relative abundance of sequence reads assigned to chloroplast 16S in each sample based on location inside, or outside (building exterior) of classrooms. “O” = outside window, “W” = inside window, “D” = inside door, “DD” = inside desk near door, “WD” = inside desk near window. Lowercase letters indicate statistically significant differences among samples.

**
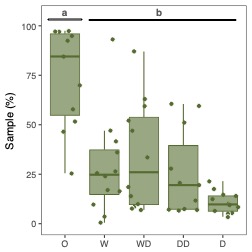
**

**Figure S4**: Bacterial communities of surfaces varied by inside/outside location, but not by inside location. NMDS ordination plot of the bacterial communities of inside samples as a function of location. Inside: Desk (D) is the desk nearest the inside classroom door, while Inside: Desk (W) is the desk nearest the window.


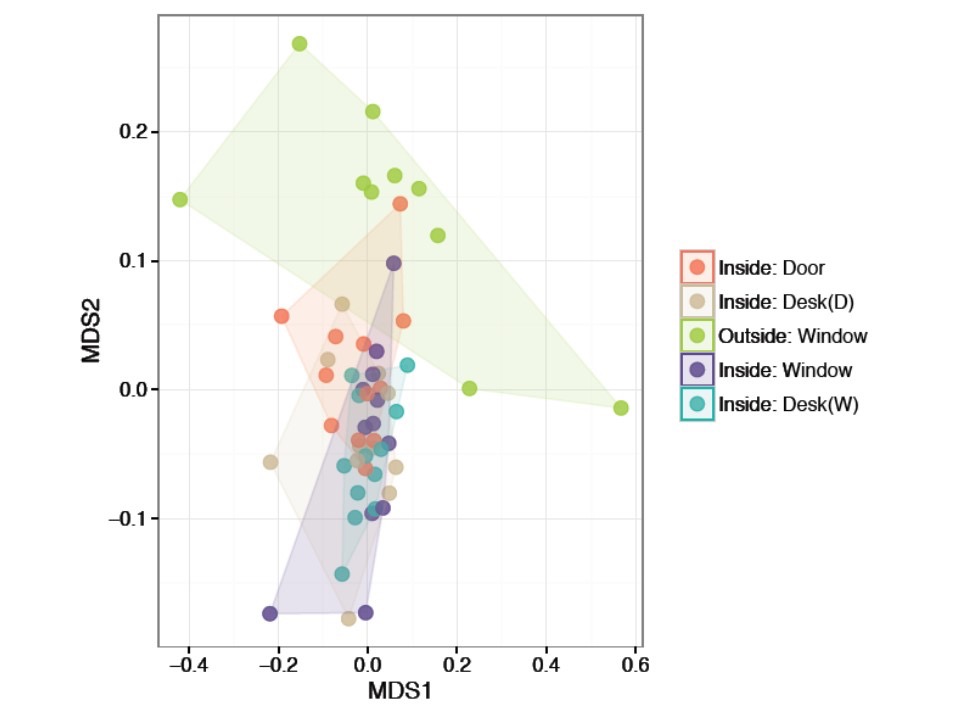


**Figure S5**. Heatmap of relative abundances of the 20 most abundant genera detected across sampled locations. “O” = outside window, “W” = inside window, “D” = inside door, “DD” = inside desk near door, “WD” = inside desk near window. Color scaled by genus.

**Figure S6.** Differentially enriched OTUs in high contact surface communities (desks, n=19) versus low contact surface communities (door trim, n=11) across classrooms as determined by a DESeq2 negative binomial Wald test with FDR corrections. Twenty-three OTUs were significantly enriched across sample types. Notably, OTUs assigned to taxa frequently found in human saliva and skin (e.g., taxa in the families Streptococcaceae, Micrococcaceae, and Moraxellaceae) were enriched in bacterial communities on high contact surfaces.


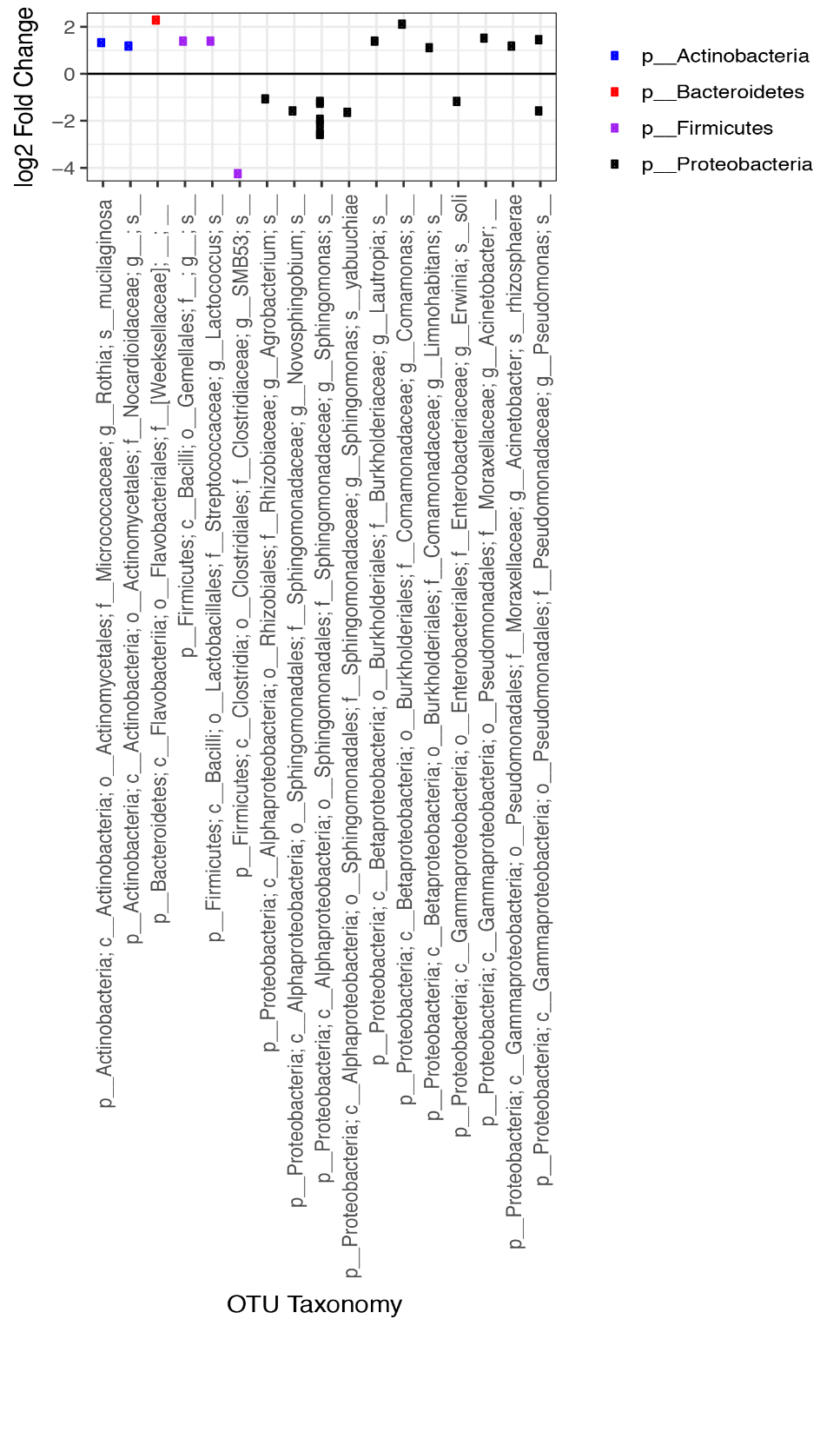


**Table S1.**

List of putative contaminant OTUs found in the negative control samples that were removed from the original dataset.

OTU ID taxonomy

OTU_5911 k__Bacteria; p__Proteobacteria; c__Alphaproteobacteria; o__Caulobacterales; f__Caulobacteraceae; g__Mycoplana; s__

OTU_209 k__Bacteria; p__Proteobacteria; c__Alphaproteobacteria; o__Caulobacterales; f__Caulobacteraceae; g__Mycoplana; s__

OTU_42 k__Bacteria; p__Proteobacteria; c__Betaproteobacteria; o__Burkholderiales; f__Oxalobacteraceae; g__Janthinobacterium; s__lividum

OTU_91 k__Bacteria; p__Proteobacteria; c__Gammaproteobacteria; o__Pseudomonadales; f__Moraxellaceae; g__Acinetobacter; s__

OTU_3565 k__Bacteria; p__Proteobacteria; c__Alphaproteobacteria; o__Caulobacterales; f__Caulobacteraceae; g__Asticcacaulis

OTU_3618 k__Bacteria; p__Proteobacteria; c__Gammaproteobacteria; o__Pseudomonadales; f__Pseudomonadaceae; g__Pseudomonas; s__veronii

OTU_73 k__Bacteria; p__Proteobacteria; c__Gammaproteobacteria; o__Pseudomonadales; f__Pseudomonadaceae; g__Pseudomonas; s__veronii

OTU_227 k__Bacteria; p__Proteobacteria; c__Gammaproteobacteria; o__Enterobacteriales; f__Enterobacteriaceae; g__; s__

OTU_40 k__Bacteria; p__Proteobacteria; c__Gammaproteobacteria; o__Xanthomonadales; f__Xanthomonadaceae; g__Stenotrophomonas; s__

OTU_1768 k__Bacteria; p__Proteobacteria; c__Alphaproteobacteria; o__Caulobacterales; f__Caulobacteraceae; g__Mycoplana; s__

OTU_4484 k__Bacteria; p__Proteobacteria; c__Gammaproteobacteria; o__Pseudomonadales; f__Pseudomonadaceae; g__Pseudomonas; s__veronii

OTU_53 k__Bacteria; p__Proteobacteria; c__Alphaproteobacteria; o__Caulobacterales; f__Caulobacteraceae; g__Mycoplana; s__

OTU_228 k__Bacteria; p__Proteobacteria; c__Betaproteobacteria; o__Burkholderiales; f__Comamonadaceae; g__Methylibium; s__

OTU_1507 k__Bacteria; p__Proteobacteria; c__Gammaproteobacteria; o__Xanthomonadales; f__Sinobacteraceae; g__; s__

OTU_2385 k__Bacteria; p__Proteobacteria; c__Gammaproteobacteria; o__Xanthomonadales; f__Xanthomonadaceae; g__Stenotrophomonas; s__

OTU_2061 k__Bacteria; p__Fusobacteria; c__Fusobacteriia; o__Fusobacteriales; f__Leptotrichiaceae; g__Sneathia; s__

OTU_5951 k__Bacteria; p__Proteobacteria; c__Gammaproteobacteria; o__Enterobacteriales; f__Enterobacteriaceae; g__Trabulsiella; s__

OTU_1951 k__Bacteria; p__[Thermi]; c__Deinococci; o__Deinococcales; f__Deinococcaceae; g__Deinococcus; s__

OTU_5294 k__Bacteria; p__Actinobacteria; c__Actinobacteria; o__Actinomycetales

OTU_2287 k__Bacteria; p__Proteobacteria; c__Deltaproteobacteria; o__Myxococcales; f__0319-6G20; g__; s__

OTU_2440 k__Bacteria; p__Cyanobacteria; c__4C0d-2; o__MLE1-12; f__; g__; s__

**Table S2.**

Table of statistical results at different sequencing depths per sample.

| **Statistical comparison** | **Sequencing depth** | **Total samples included in analysis** | **Test** | **Test statistic** | **p-value *** |
| --- | --- | --- | --- | --- | --- |
| Inside vs outside window samples | 200 | 22 | Adonis | F=2.209, R^2^=0.10 | **0.001** |
|  | 1000 | 17 | Adonis | F=2.6423, R^2^=0.15 | **0.004** |
| Inside samples by center and class. Factor: Center | 200 | 47 | Adonis | F=1.47, R^2^=0.15 | **0.001** |
|  | 1000 | 41 | Adonis | F=1.91, R^2^=0.21 | **0.001** |
| Inside samples by center and class. Factor: Class | 200 | 47 | Adonis | F=1.16, R^2^=0.14 | **0.005** |
|  | 1000 | 41 | Adonis | F=1.19, R^2^=0.04 | **0.003** |
| Desks versus door trim (High vs. low contact) | 200 | 35 | Adonis | F=1.43, R^2^=0.04 | **0.005** |
|  | 1000 | 30 | Adonis | F=1.54, R^2^=0.052 | **0.006** |
| Inside by locations | 200 | 47 | Adonis |  | ns |
|  | 1000 | 41 | Adonis |  | ns |
| Correlation between inside communities and lux | 200 | 47 | Mantel |  | ns |
|  | 1000 | 41 | Mantel |  | ns |
| Correlation between inside communities and child density | 200 | 47 | Mantel | r=0.19 | **0.04** |
|  | 1000 | 41 | Mantel | r=0.251 | **0.022** |

* ns = not statistically significant

**Table S3.**

Mantel tests based on Pearson’s product-moment correlation of Bray-Curtis distance metrics of lux and child occupant density for inside samples (inside window, desk by window, desk by door, inside door trim) across classrooms (samples: n=47):

| **Metric: bacterial beta diversity by classroom** | **p-value** | **Mantel statistic (r)** |
| --- | --- | --- |
| Occupant Density | 0.04* | 0.19 |
| **Metric: bacterial beta diversity by sampling location** | | |
| Lux | 0.25 | 0.06 |
